# EMANCIPATORY VISIONS: USING VISUAL METHODS TO CO-CONSTRUCT KNOWLEDGE WITH OLDER ADULTS

**DOI:** 10.1093/geroni/igac059.1648

**Published:** 2022-12-20

**Authors:** Jarmin Yeh, Laurent Reyes, H Shellae Versey

**Affiliations:** University of California, San Francisco, San Francisco, California, United States; UC Berkeley, Berkeley, California, United States; Fordham University, Bronx, New York, United States

## Abstract

Research is a political activity. Researchers are responsible for theories and methods used to explore, explain, or ignore injustices. A need exists for developing new tools and pathways of knowledge based on experiences, language, and intellect of older adults from Black, Indigenous, and People of Color communities. This presentation argues for the utility of visual methods in critical qualitative research as a medium that allows researchers and participants to co-construct knowledge. Lessons learned about implementing projects using visual methods from the intersectional standpoint of the authors – three younger women of color – will be discussed. Promises and complexities of navigating interpersonal dynamics, decolonizing knowledge-production, and scaling visual methods on multiple levels will be elucidated. Collectively, we argue that visual methods are rigorous for subverting power dynamics rooted in extractive research practices, and provide a vehicle for community-engaged participatory action research that has potential to advance social justice in gerontology.

